# Parametrizing the Spatial Dependence of ^1^H NMR Chemical Shifts in π-Stacked Molecular Fragments

**DOI:** 10.3390/ijms21217908

**Published:** 2020-10-24

**Authors:** Jiří Czernek, Jiří Brus

**Affiliations:** Institute of Macromolecular Chemistry, Czech Academy of Sciences, Heyrovsky Square #2, 16206 Prague, Czech Republic; brus@imc.cas.cz

**Keywords:** noncovalent interactions, proton NMR, intermolecular stacking, GIAO, GIPAW

## Abstract

Most recently a renewed interest in several areas has arisen in factors governing the ^1^H NMR chemical shift (^1^H CS) of protons in aromatic systems. Therefore, it is important to describe how ^1^H CS values are affected by π-stacking intermolecular interactions. The parametrization of radial and angular dependences of the ^1^H CS is proposed, which is based on conventional gauge-independent atomic orbital (GIAO) calculations of explicit molecular fragments. Such a parametrization is exemplified for a benzene dimer with intermonomer vertical and horizontal distances which are in the range of values often found in crystals of organic compounds. Results obtained by the GIAO calculations combined with B3LYP and MP2 methods were compared, and revealed qualitatively the same trends in the ^1^H CS data. The parametrization was found to be quantitatively correct for the T-shaped benzene dimers, and its limitations were discussed. Parametrized ^1^H CS surfaces should become useful for providing additional restraints in the search of site-specific information through an analysis of structurally induced ^1^H CS changes.

## 1. Introduction

The proton nuclear magnetic resonance (^1^H NMR) is a crucial technique for solving a vast variety of chemical, physical, and biological problems in solution [1] and solid phases [2]. Some of those problems concern the dispersive interactions [3] between aromatic moieties and the role they play in various phenomena (see references [4,5,6,7] for the most recent examples). In such studies, the key parameter is the ^1^H chemical shift (^1^H CS) perturbation caused by the presence of aromatic fragments in the vicinity of an investigated proton [8,9]. It should be noted that the ^1^H CS of protons in aromatic molecules has received considerable interest, as most recently summarized in references [10,11]. Therefore, it is desirable to study factors governing the ^1^H CS values in molecules which exhibit C–H/π aromatic stacking interactions. Here, conventional gauge-independent atomic orbital (GIAO) calculations (see Materials and Methods) are carried out for the proton sites in two types of stacked molecular dimers. The first type are benzene dimers which serve for the purpose of a parametrization of the ^1^H CS landscape, as detailed in Section 2.1, and for checking the precision of the proposed parametrization. The second type of investigated dimers are those taken from crystals of midsized organic molecules [12,13] (see Section 2.2). For these systems, accurate ^1^H CS data are available from the solid-state NMR (SSNMR) measurements [12,14], and hence they are used in reliability considerations. In particular, the GIAO ^1^H CS estimates for the dimers are confronted with experiment and with results of the gauge-including projector augmented wave (GIPAW) calculations performed in a plane-wave density functional theory (PW DFT) scheme for periodic structures of aforementioned crystals [15]. In this way, it has been shown that it should be possible to successfully apply the parametrization process to other ligands and to configurations relevant for binding of those ligands. At the same time, an inherent limitation of this approach should be noted, which is the presence of a generally shaped contour line for a given ^1^H CS value, as discussed in Section 3 and visualized. However, the parametrized ^1^H CS surfaces are expected to be applied together with other structural information provided by NMR and possibly diffraction measurements, or by advanced computational methods [16], which would constrain stacking fragments in the correct spatial arrangement compatible with some specific ^1^H CS value. Importantly, a set of ^1^H CS values could, then, be used to elucidate structures of complex systems, for instance, polydopamine [17,18,19,20]. It should also be noted that the ^1^H CS surfaces can be obtained with an inclusion of solvation at the interacting sites [21] in order to properly describe binding between organic ligands and models of large biologically active molecules in solution.

## 2. Results

### 2.1. Development of the Proton Chemical Shift (^1^H CS) Surface

**Figure 1:**
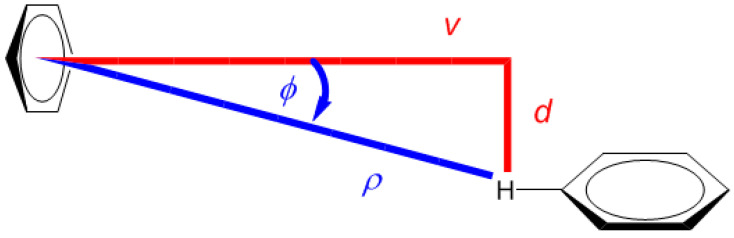
Schematic representation of the benzene dimer together with the Cartesian (*v*, *d*) and polar (*ρ*, *ϕ*) coordinate systems used in this work.

The energy parameters of clusters of benzene in various media have been intensely studied [22,23]. The potential energy surface (PES) of the gas-phase benzene dimer is quite well understood [24]. Here, for the purpose of describing the landscape of the ^1^H CS values, the T-shaped dimer in “*C_2v_* over atom” configuration was adopted from the work of Head-Gordon at al. [25] (the energetics are discussed in Section 2.3 and put into context of other configurations). Using this structure, the grid consisting of 7 × 7 points was constructed by simultaneously varying two parameters, without any relaxation of the geometry. These parameters were (i) *v*, the vertical separation between the investigated proton and the center of the other monomer and (ii) *d*, the lateral displacement in the plane parallel to the monomer not containing that proton (see Figure 1). It should be mentioned that one of the grid points corresponded to the geometry directly taken from reference [25], with *v* = 2.302 Å and *d* = 0.0 Å, and that all structures with nonzero values of *d* were of *C_s_* symmetry. For the points from intervals of *v* between 2.002 and 3.402 Å and of *d* between 0.0 and 1.5 Å, the ^1^H CS isotropic chemical shielding value of the investigated proton was provided by the GIAO and GIAO and Becke’s three-parameter together with Lee–Yang–Parr functionals (GIAO-MP2)/6-311++G(2d,2p), and GIAO and Becke’s three-parameter, Lee–Yang–Parr (GIAO-B3LYP)/6-311++G(2d,2p) approaches (see Materials and Methods). These two methods were successfully applied to investigate trends in the chemical shielding [26,27,28]. Estimates of the ^1^H CS values, denoted as *δ*, were obtained using the ^1^H isotropic chemical shielding calculated by the corresponding method for a proton of tetramethylsilane molecule optimized at the MP2/aug-cc-pVTZ level, as detailed in Section 4.

An attempt was made to analytically describe the dependence of *δ* upon the variables *v* and *d*. At first, however, geometries were expressed in the polar coordinates (*ρ*, *ϕ*), also depicted in Figure 1. Numerical experiments were, then, performed in this coordinate system using relevant toolboxes of Matlab^®^, and it was found that both sets of input *δ* data could be successfully fitted to a relatively simple functional form given by Equation (1). Subsequently, the fitting was repeated in the (*v*, *d*) coordinate system while employing “e04fcf” subroutine from NAG^®^ Library (related f90 program also used “lsqgrd” subroutine to check that the gradients of $\delta_{\mathrm{model}}$, taken with respect to {A, B, C, D, E, F} parameters of Equation (1), were sufficiently close to zero). The resulting parameter values were considered to be final. They are listed in Supplementary Materials Table S1, and both sets of 49 *δ* points are available from Table S4. The sole idea behind finding this analytic expression for *δ* is to obtain a formula which is sufficiently accurate for numerically describing the ^1^H CS surfaces, and Equation (1) should not be interpreted in any other way. It follows from a comparison of the GIAO-MP2 and GIAO-B3LYP surfaces that these methods provided qualitatively the same ^1^H CS landscape. This is significant, since the GIAO-B3LYP/6-311++G(2d,2p) approach, contrary to its MP2 counterpart, can be routinely applied to large molecular fragments, possibly containing more than one hundred atoms. Satisfactory precision of the fitting is also important, i.e., the maximum absolute deviation, average absolute deviation, residual norm, and *x*^2^ are 0.28 ppm, 0.10 ppm, 0.83 ppm, and 0.1247, respectively, for the GIAO-MP2 data, while these values accordingly amount to 0.32, 0.09, 0.61 ppm and 0.0993 for the GIAO-B3LYP data.

(1) $$\delta_{\mathrm{model}}\left(\rho ,\phi ;A,\;B,\;C,\;D,\;E,\;F\right)=A+B\;\sin\left(\frac{\phi -F}{C}\right)\sin\left(\pi \frac{\rho -E}{D}\right)\;\mathrm{parametrized}\;\mathrm{for}\;\rho \in \langle 2.0020;3.7189\rangle \;Å,\;\phi \in \langle 0.0;0.6428\rangle \;\mathrm{rad}$$

**Figure 2:**
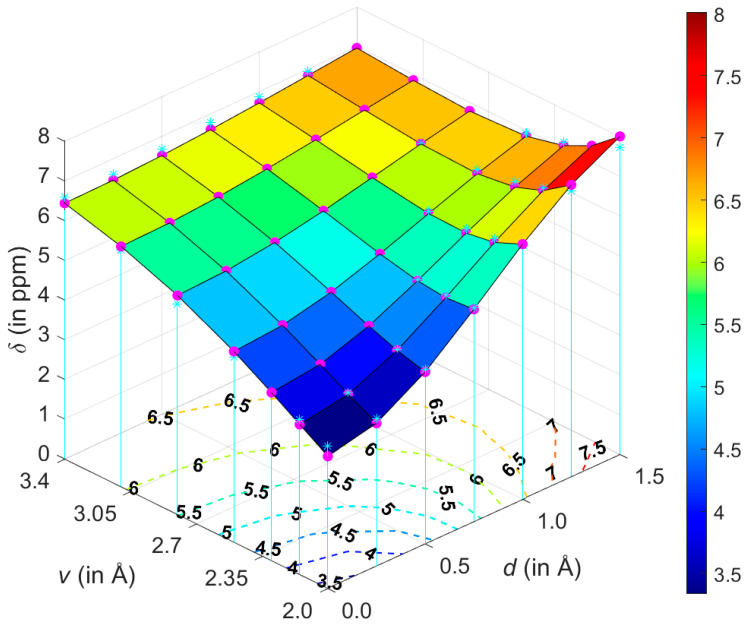
The geometry dependence of the GIAO-MP2/6-311++G(2d,2p) predicted ^1^H chemical shifts (magenta circles) and their parametrization (cyan stars are shown at the grid points) obtained for the T-shaped benzene dimer.

Figure 2 graphically presents an example ^1^H CS surface and its fairly complex curvature and other issues are discussed in Section 3, while reliability of the model is addressed below.

This description of a spatial arrangement of benzene monomers using only two geometry variables is, of course, simplified. Hence, it is of importance to assess how accurate the ^1^H CS parametrization would be if applied to the benzene dimer configurations with mutual orientation of planes other than the one shown in Figure 1. This assessment was performed for the tilted T-shaped structures from [29], which are available from the BEGDB database [30] (their dissociation curve is analyzed in Section 2.3). The *v*, *d* values of five of those geometries fall into the parametrized interval, and therefore their coordinates were used to obtain the ^1^H CS from the model and directly from the GIAO calculations. The predicted ^1^H chemical shielding data are summarized in Table S2 and show only small differences between the parametrized values and their counterparts provided explicitly by quantum chemical calculations (maximum discrepancy amounts to 0.28 ppm and is found for the MP2 data of the structure with *v* = 2.6595 Å and *d* = 0.1361 Å). Moreover, these datasets exhibit the same geometry dependence, namely, the ^1^H chemical shielding values decrease with an increase in *v* and *d* in this investigated region. The above two findings show that the presented model also works well for the benzene dimers featuring interplanar angles which have not been included in the parameterization. Therefore, it seems that it might be possible to use only two spatial coordinates to reliably quantify how π-stacking interactions affect the ^1^H CS of protons in aromatic systems in general. However, it should be kept in mind that this model relies on the ability of quantum chemical calculations to accurately describe the pair interactions influencing the ^1^H CS value in a molecular cluster. Hence, in Section 2.2, this ability is evaluated for organic solids whose structures pose additional challenges due to crystal packing and due to heteroatoms or substituents present in the aromatic ring that also contains the investigated proton.

### 2.2. Validation of the Dimer Model 

**Figure 3:**
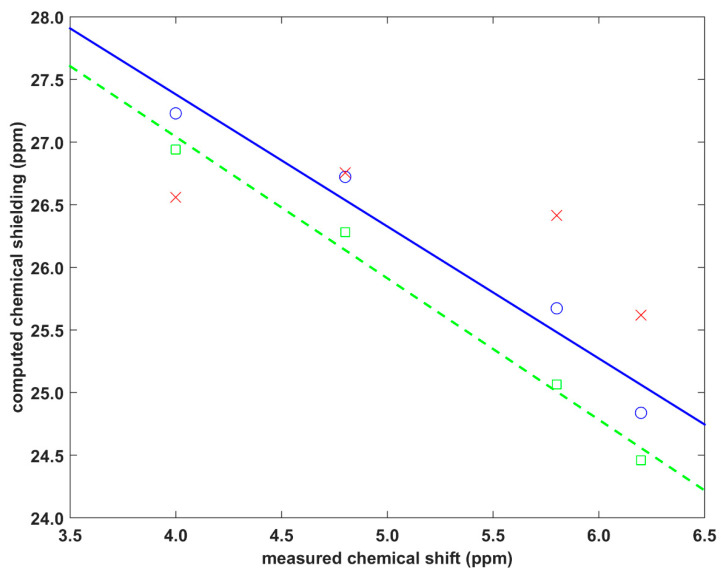
Comparison between theory and experiment for the proton solid-state nuclear magnetic resonance (^1^H SSNMR) parameters of two molecular crystals specified in the text. Green squares, blue circles, and red crosses pertain to the GIPAW-PBE, GIAO-B3LYP, and parametrized data, respectively (the green dashed line is y = −1.292*x + 31.56 ppm, while the blue straight line is y = −1.054*x + 31.60 ppm).

Older research on the ^1^H CS of stacked proton sites in molecular crystals has been presented in the excellent review article [31]. Here, two solid phase systems are analyzed, which have been carefully studied by the groups of Kentgens [14] and Brown [12], and thus they provide the benchmark data. Their data are considered to be fully reliable and cover a relatively large interval of pertinent ^1^H CS values (see Table 1). The first system considers the protons, numbered H10′ and H11′ and shown in Figure 11b of [14], of the crystalline isocyanoalanyl carbazole amid [13]. The second system concerns the 1:1 cocrystal of dithianon and pyrimethanil with protons numbered H2 and H25 and shown in Figure 5 of [12]. Experimental and theoretical data are collected in Table 1 (the following values of the ^1^H absolute isotropic chemical shielding of a proton in tetramethylsilane were used for referencing: 30.8868 ppm for GIPAW and Perdew-Burke-Erzerhof (GIPAW-PBE) and 31.8308 ppm for GIAO-B3LYP, respectively). The investigated protons are practically unaffected by the C–H…X hydrogen bonding, which is not the case, for example, for aromatic protons in the well-known 1:1 cocrystal of indomethacin and nicotinamide [32,33], or in some systems previously studied by this authors, in particular, L-tyrosine hydrochloride [34]. Consequently, the investigated protons are supposed to probe an influence of the C–H/π stacking only and are used for further testing of the present approach (see Figure 3; computational estimates of the ^1^H CS are plotted against experimental values in Figure S2). For this type of environment, it was ascertained that the GIAO-B3LYP/6-311++G(2d,2p) dimer calculations could provide accurate ^1^H chemical shielding values, i.e., the standard deviation of residuals and adjusted *R*^2^ of the corresponding linear regression are 0.21 ppm and 0.93, respectively. Accordingly, these results amount to 0.12 ppm and 0.98 for the periodic GIPAW calculations which are, thus, highly successful, as was anticipated on the basis of previous work on structurally similar systems, for instance, naproxen [35,36]. It has to be mentioned, however, that large errors of the PW DFT predicted ^1^H CS may occur for hydroxyl hydrogen-bonded sites [37,38,39], but those discrepancies are caused mainly by the proton dynamics [40,41,42] that is unlikely to be significant in the molecular crystals studied here. Nevertheless, the current results validate the approach which applies the GIAO-B3LYP calculations of dimers for describing the spatial dependence of the ^1^H CS in fragments affected by π-stacking in molecular solids.

Corresponding results obtained from the model expressed by Equation (1) are also plotted in Figure 3. Their quality is generally poor, as the parametrization was carried out using the benzene dimer, not for fragments actually present in the investigated crystals. However, an inspection of these results indicates that the parameters of Equation (1) may be transferable to structurally similar residues. This can be seen for the proton site involved in the C–H/π interaction between fused (and otherwise unsubstituted) benzene rings. Namely, the H11′ site of the isocyanoalanyl carbazole amid structure [13] is located on one such ring (denoted as “P” in [14]) of a carbazole unit, and is stacked by another benzene ring (also “P”) of the neighboring molecule. As shown in Figure 3, at the ^1^H CS of 4.8 ppm, the difference between the parametrized result and the GIAO-B3LYP value is minimal (0.04 ppm). The contacts of the remaining protons (H11′, H2, and H25 of the structures specified above) involve substituted rings and are not detailed here. Separate parametrizations would be needed in order to accurately describe the ^1^H CS in these moieties. It should be kept in mind that the presented analysis is implicitly influenced by the ^1^H CS measurement uncertainties. Those uncertainties can be expected to be about 0.2 ppm, but they could be reduced, in particular, by using ultrafast magic-angle spinning SSNMR techniques [43,44,45], and at very high magnetic fields.

### 2.3. Dimerization Energy Considerations

**Figure 4:**
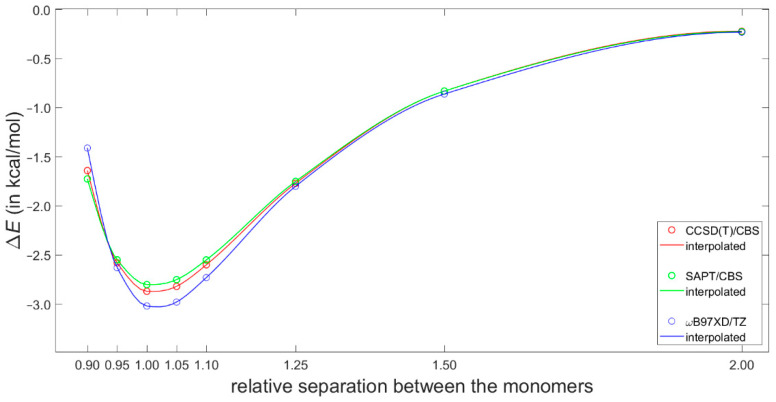
Binding energies of the tilted T-shaped benzene dimer described in the text.

**Figure 5:**
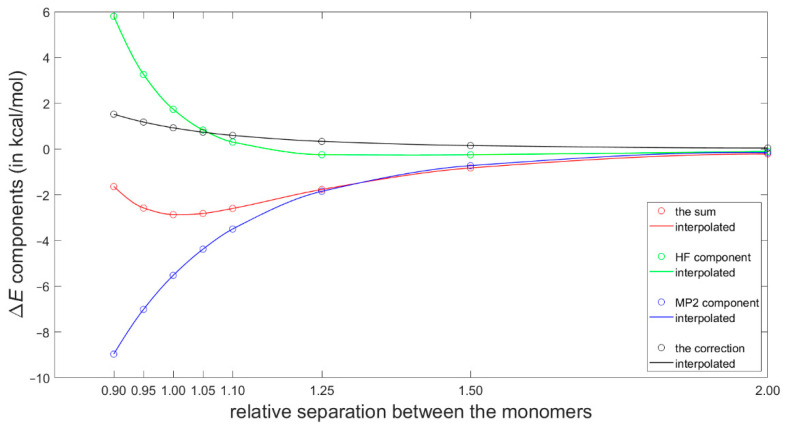
The CCSD(T)/CBS interaction energy components at points along the dissociation curve of the tilted T-shaped benzene dimer.

Most recently, Platzer et al. [5] discussed a possible connection between increasing binding enthalpy and a higher ^1^H CS perturbation for certain geometries of fragments involved in CH–π interactions in protein–ligand complexes. Specifically, they scanned the PES of a T-shaped benzene dimer using the ωB97X-D/cc-pVTZ dispersion corrected DFT approach (see Materials and Methods for referencing of this and the subsequent computational techniques), which had been previously shown to reliably describe the sandwich configuration [46]. Of practical significance is the fact that the ωB97X-D/cc-pVTZ interaction energy can be obtained using a tiny fraction of computational resources needed to estimate the corresponding “gold standard” coupled cluster singles and doubles with iterative inclusion of triples (CCSD(T))/complete basis set (CBS) value (such a value would at present be inaccessible for systems containing more than about 60 atoms) [47]. Moreover, the ωB97X-D method combined with a midsized basis set have recently been used in important investigations of stacking [16,48]. Hence, the ωB97X-D/cc-pVTZ approach was applied here to calculate interaction energies at the grid points for the ^1^H CS calculations described above. These interaction energies are provided in Table S4 and graphically presented in Figure S1. They are assumed to be quite accurate based on aforementioned references and also based on a high quality of the data obtained for the dissociation curve of the tilted T-shaped benzene dimer (see Figure 4 and Table S3). Namely, for its geometries reported in [29] and deposited in the BEGDB database [30], the ωB97X-D/cc-pVTZ results were compared to fully reliable CCSD(T)/CBS data and to their DFT-based symmetry-adapted intermolecular perturbation theory (DFT-SAPT)/CBS counterparts. Figure 4 shows only insignificant differences between the ωB97X-D/cc-pVTZ and the two sets of CBS-extrapolated values. The outstanding performance of the DFT-SAPT computational protocol, specified in Section 4, and whose results closely match the CCSD(T)/CBS points of the investigated curve are also noteworthy. As for the CCSD(T)/CBS values, they were reconstructed according to the description provided in [29] in order to examine their breakdown into the $\Delta E_{\mathrm{HF}}^{\mathrm{aQZ}}$, $\Delta E_{\mathrm{MP}2}^{\mathrm{extrap}.}$, and $\Delta E_{\mathrm{CCSD}\left(T\right)-\mathrm{MP}2}^{\mathrm{correction}}$ components (see Equation (3) in Section 4). This breakdown is shown in Figure 5 and illustrates a delicate balance of contributions to the interaction energy.

## 3. Discussion

**Figure 6:**
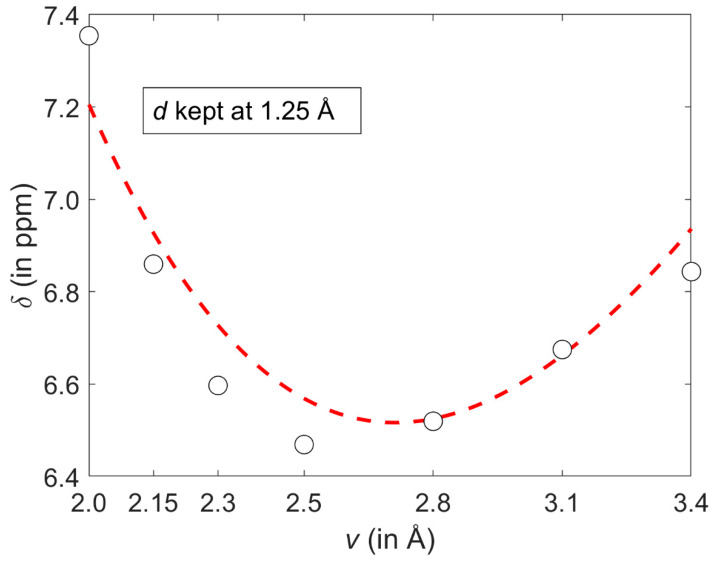
A cut through the GIAO-MP2/6-311++G(2d,2p) ^1^H chemical shift (CS) surface discussed in the text. Values obtained from regular calculations and from the corresponding parametrization are shown as open black circles and the dashed red line, respectively.

The GIAO-MP2 and GIAO-B3LYP methods were combined with the 6-311++G(2d,2p) basis set and applied to a relatively large interval of radial and angular orientations of the benzene dimer (both parametrizations are provided as Matlab m-files in Supplementary Materials). It is stressed that a majority of these orientations fulfil the commonly used criteria for aromatic C–H/ π bonding. Specifically, the Brandl–Weiss geometric system for identification of C–H/π interactions employs in a lateral dimension the coordinate denoted as $d_{H_{p}-X}$ [49] that is numerically equal to the coordinate *d* used here. In structural database searches, a cut-off value for $d_{H_{p}-X}$ of “1.0 or 1.2 Å for different sized π -acceptor systems“ is typically used [50] and would have covered most of the grid points if it had been applied in this work. Nevertheless, the GIAO calculations revealed a complicated landscape of the ^1^H CS values of the proton directly involved in the C–H/π interaction. These values do not change monotonically with increasing intermonomer separation for all lateral displacements considered here. An illustration of this non-monotonic behavior is provided in Figure 6 which shows a slice of the ^1^H CS surface taken at *d* = 1.25 Å. Using the parametrization expressed by Equation (1), a minimum of $\delta_{\mathrm{model}}$ with respect to the vertical distance *v* can be easily obtained by taking the partial derivative and, after the (*ρ*, *ϕ*) → (*v*, *d*) coordinate transformation, solving for zero the right hand side of Equation (2) with *d* fixed at 1.25 Å.

(2) $$\frac{\partial }{\partial \phi }\delta_{\mathrm{model}}\left(\rho ,\phi ;A,\;B,\;C,\;D,\;E,\;F\right)=-\left[B\;\pi \cos\left(\frac{\pi \left(F-\phi \right)}{C}\right)\sin\left(\frac{\pi \left(E-\rho \right)}{D}\right)\right]/C$$

**Figure 7:**
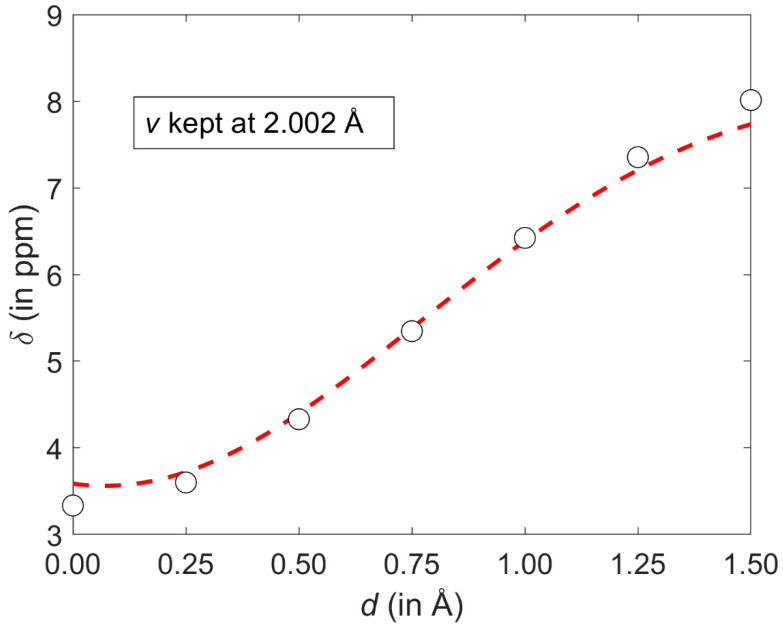
A cut through the GIAO-MP2/6-311++G(2d,2p) ^1^H CS surface discussed in the text. Values obtained from regular calculations and from the corresponding parametrization are shown as open black circles and the dashed red line, respectively.

At such a minimum point, the ^1^H CS is 6.5170 ppm and *v* is 2.7133 Å, apparently in agreement with Figure 6. For *d* = 1.25 Å and ***v*** = 2.7133 Å, the second derivative $\frac{\partial^{2}}{\partial \phi^{2}}\delta_{\mathrm{model}}$ has a negative value (namely, −1.6529 ppm/Å^2^), of course confirming that the examined slice is convex. However, the ^1^H CS grows monotonically with increasing displacement in the whole interval of investigated vertical distances (an example of this dependence is given in Figure 7). As a consequence of completely different profiles in *v* and *d* dimensions, contour lines have an irregular shape, which would not be the case if simple models were applied (such models were carefully compared in a relatively recent study) [51]. Several contour lines are visualized in Figure 2, and numerical examples now follow. For the lowest vertical separation considered here (*v* = 2.002 Å), it is immediately found through Equation (1) that the particular values of ^1^H CS of [5.5, 6.0, and 6.5 ppm] are, respectively, reached at *d* = 0.7786, 0.9005, and 1.0305 Å. Then, using these displacement values, the ^1^H CS of 5.5, 6.0, and 6.5 ppm are located at *v* = 2.3648, 2.7317, and 3.0944 Å, respectively. This analysis explicitly shows that the same ^1^H CS value may occur in vastly different spatial arrangements of stacked molecules. Additional information would obviously be needed to resolve such ambiguities during a structure determination process, analogously to the requirement for neutron diffraction data in distinguishing hydrogen bond networks of some polymorphs [52].

## 4. Materials and Methods

The standard second-order Møller–Plesset (MP2) approach and the standard Becke’s three-parameter, Lee-Yang–Parr (B3LYP) combination of DFT functionals were used. In the chemical shielding calculations, these methods were combined with the standard 6-311++G(2d,2p) basis set and with the GIAO strategy to overcome the gauge problem [53,54]. Interaction energies corrected for the basis set superposition error by the counterpoise (CP) scheme [55] were obtained using the empirically corrected ωB97X-D DFT functional [56] and the standard cc-pVTZ (correlation-consistent polarized valence triple-ζ) basis set. In the full geometry optimization of tetramethylsilane, the MP2 method was combined with the standard aug-cc-pVTZ (the cc-pVTZ augmented with diffuse functions) basis set, and *T_d_* symmetry was imposed. All these calculations were carried out using the Gaussian 09 suite of codes [57].

Crystal structures [12,13] served as input for periodic DFT calculations which adopt the pseudopotential scheme [58,59,60] implemented in the CASTEP 16.1 program [60]. Unit-cell parameters of these structures were kept at experimental values, while atomic positions were optimized with respect to the crystal-lattice energy that was approximated using the Perdew–Burke–Erzerhof (PBE) DFT functional [61]. For resulting geometries, the chemical shielding was predicted by applying the GIPAW method [62,63] and the PBE functional. In all CASTEP calculations, the settings were consistent with “Fine” accuracy level of the Materials Studio 2019 software [64]. In particular, the PW cut-off value was 42.0 Ry. The ultrasoft on-the-fly generated pseudopotentials were adopted [65].

The CCSD(T) (coupled cluster singles and doubles with iterative inclusion of triples) interaction energies extrapolated to the CBS (complete basis set) limit, $\Delta E_{\mathrm{CCSD}\left(T\right)}^{\mathrm{CBS}}$, were estimated using Equation (3)

(3) $$\Delta E_{\mathrm{CCSD}\left(T\right)}^{\mathrm{CBS}}=\Delta E_{\mathrm{HF}}^{\mathrm{aQZ}}+\Delta E_{\mathrm{MP}2}^{\mathrm{extrap}.}+\Delta E_{\mathrm{CCSD}\left(T\right)-\mathrm{MP}2}^{\mathrm{correction}}$$

The $\Delta E_{\mathrm{HF}}^{\mathrm{aQZ}}$ term is the interaction energy obtained at the Hartree–Fock (HF) level using the aug-cc-pVQZ basis set. The $\Delta E_{\mathrm{MP}2}^{\mathrm{extrap}.}$ denotes the MP2 correlation energy contribution, which was obtained using the aug-cc-pVTZ and aug-cc-pVQZ values by their extrapolation that employed the scheme of Halkier et al. (Equation (7) in [66]). The $\Delta E_{\mathrm{CCSD}\left(T\right)-\mathrm{MP}2}^{\mathrm{correction}}$ component, which aims at correctly approximating higher-order correlation energy contributions to the interaction energy, was computed using the aug-cc-pVDZ basis set in the underlying CCSD(T) and MP2 calculations. The CP correction was applied throughout.

The DFT-based SAPT (symmetry-adapted intermolecular perturbation theory) [67] calculations (abbreviated as DFT-SAPT) were performed in the density-fitting variant [68]. The procedure described in [69] was followed except for an extrapolation of the interaction energy components. Namely, in this work all the components were extrapolated to their CBS limit and summed up, while Hesselmann et al. [69] extrapolated only the second-order dispersion contributions. The Molpro version 2008.1 [70] was used to obtain energies leading to the CCSD(T)/CBS and DFT-SAPT/CBS results.

## Figures and Tables

**Table 1:** Structural and spectral parameters of investigated solid-phase systems. In this table, the measured chemical shift is denoted as δ, and its theoretical estimate as δ’. See the text for details.

Crystal	Site	*v* (in Å)	*d* (in Å)	δ (in ppm)	*σ*/δ’ Periodic (in ppm)	*σ*/δ’ Cluster (in ppm)	*σ*/δ’ Fit (in ppm)
Dithianon–pyrimethanil	H25	2.5238	0.5056	4.0	26.9417/3.9	27.2285/4.6	26.5599/5.3
	H2	2.6975	0.9521	6.2	24.4601/6.4	24.8389/7.0	25.6178/6.2
The isocyanide	H10′	2.6009	0.1846	4.8	26.2797/4.6	26.7231/5.1	26.7550/5.1
	H11′	2.7557	0.2720	5.8	25.0655/5.8	25.6729/6.2	26.4137/5.4

## Supplementary Materials

The following are available online at https://www.mdpi.com/1422-0067/21/21/7908/s1, Table S1: Values of parameters in Equation (1), Table S2: The ^1^H chemical shielding data for the tilted T-shaped dimer of benzene, Table S3: Interaction energies of the tilted T-shaped dimer of benzene, Table S4: Predicted values of the ^1^H CS and of the ωB97X-D/cc-pVTZ interaction energy at 49 grid points, Figure S1: A plot of the ωB97X-D/cc-pVTZ interaction energies, Figure S2: A plot of measured and computed ^1^H chemical shifts of selected protons in two molecular crystals; script files ‘f_b3lyp.m’ and ‘f_mp2.m’ to obtain an estimate of the ^1^H CS on parametrized *δ* (*v*, *d*) surface.
